# DB-LIO: Database-Driven LiDAR–Inertial Odometry for Memory-Bounded Persistent Mapping

**DOI:** 10.3390/s26103061

**Published:** 2026-05-12

**Authors:** Hun-Hee Kim, Ho-Hyun Kang, Dong-Hee Noh, Hea-Min Lee

**Affiliations:** 1 AI Application Research Center, Korea Electronics Technology Institute (KETI), Jeonju 54853, Republic of Korea; sdgo2363@keti.re.kr (H.-H.K.); khh_96@keti.re.kr (H.-H.K.); dhee.noh@keti.re.kr (D.-H.N.); 2 Division of Electronic Engineering, Jeonbuk National University, Jeonju 54896, Republic of Korea

**Keywords:** LiDAR-SLAM, memory management, spatial indexing, LRU cache, factor graph optimization, sliding window, ROS2

## Abstract

This paper proposes DB-LIO (database-driven LiDAR-inertial odometry), a simultaneous localization and mapping (SLAM) system that addresses memory scalability challenges in extended autonomous operation. Existing LiDAR-SLAM systems accumulate keyframe history in memory, leading to O(N) growth and out-of-memory failures during extended operation. To overcome this limitation, DB-LIO introduces three core design elements. First, it proposes a spatially indexed keyframe management scheme that persistently stores keyframes in SQLite with R-Tree spatial indexing, enabling O(logN+k) spatial queries that tightly couple cache eviction with factor-graph optimization requirements—a design that ensures every keyframe potentially involved in the next optimization cycle resides in cache. Second, it presents a four-level memory bounding architecture—SLAM-engine keyframe trimming with transparent on-demand reloading, a DB-level least recently used (LRU) cache with a spatial active window, Scan Context descriptor pool bounding, and iSAM2 sliding window compaction with a sparse global anchor graph—that collectively bounds the dominant memory consumers to O(C). Third, the DB-based persistent storage enables a localization mode that can reload previously built maps—including full point clouds, six-degree-of-freedom poses, timestamps, and inter-keyframe relationships—and perform pose estimation using the stored map, which is particularly valuable for agricultural robots and other autonomous systems requiring map reuse. Experiments on a custom orchard dataset demonstrate an 81.9% reduction in memory usage compared with that of the in-memory baseline (2888 MB → 524 MB), while preserving equivalent trajectory accuracy (absolute trajectory error (ATE) root mean square error (RMSE) 0.305 ± 0.001 m vs. 0.296 m). Validation on the KITTI odometry benchmark confirms that the proposed localization mode generalizes across different LiDAR types (Livox Mid360, Velodyne HDL-64E) and environments (orchard, urban driving).

## 1. Introduction

Simultaneous localization and mapping (SLAM) is a core capability for autonomous mobile robots operating in unknown environments. Among various sensor modalities, light detection and ranging (LiDAR)-based SLAM provides accurate three-dimensional (3D) geometric information regardless of lighting conditions [1]. When combined with inertial measurement units (IMUs), LiDAR-inertial odometry (LIO) systems achieve robust state estimation even in feature-sparse or dynamic environments [2,3]. In agricultural robotics, in which robots must operate continuously across large orchards and fields [4,5], reliable SLAM under extended operation is essential for autonomous navigation tasks such as path planning and obstacle avoidance.

**Figure 1:**
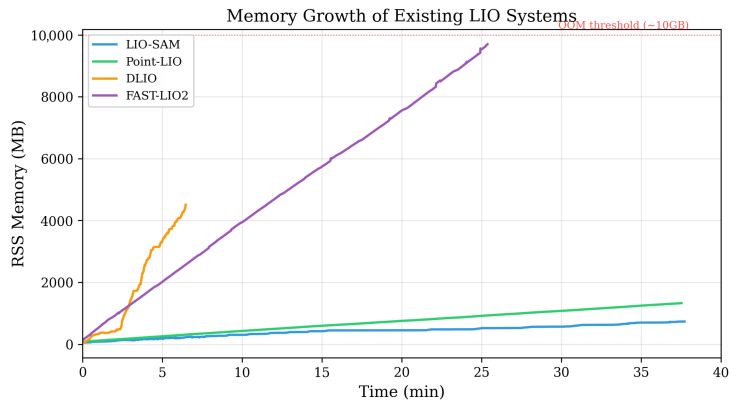
Memory usage growth over time for existing LIO systems. DLIO experienced an out-of-memory (OOM) crash and FAST-LIO2 diverged owing to rapid memory growth, demonstrating that unbounded memory accumulation prevents these systems from completing extended missions.

Unlike visual SLAM, whose low-dimensional feature vectors lend themselves naturally to database management system (DBMS) indexing, 3D LiDAR SLAM generates high-density point clouds containing hundreds of thousands of points per frame. LiDAR SLAM algorithms have traditionally relied on in-memory spatial indices such as octrees or k-dimensional (KD)-trees rather than disk-based DBMS to meet real-time tracking requirements. While this ensures real-time tracking, it introduces a fundamental scalability problem: *unbounded memory growth during extended operation*.As shown in Figure 1, existing systems such as LIO-SAM [2] use sliding windows for local map construction but accumulate all keyframe history for loop closure detection and global optimization, represented by the following data structure:

(1) $$M_{\mathrm{memory}}=\left\{\left(T_{i},P_{i}\right)|i=1,\ldots ,N\right\}$$

where $T_{i}\in SE\left(3\right)$ represents the six-degree-of-freedom (6-DoF) pose of keyframe *i*, $P_{i}\in R^{n_{i}\times 3}$ is the associated point cloud with $n_{i}$ points, and *N* is the total number of keyframes. Memory consumption grows as follows:

(2) $$M\left(N\right)=N\cdot \left(\underset{\mathrm{pose}}{\underset{︸}{\left|T\right|}}+\underset{\mathrm{point}\;\mathrm{cloud}}{\underset{︸}{\bar{n}\cdot s_{p}}}\right)=O\left(N\right)$$

where $\bar{n}$ is the average number of points per keyframe, and $s_{p}$ is the size of each point (typically 16 bytes for X, Y, Z, and Intensity (XYZI) format). Based on the growth rate observed in our outdoor mapping experiments (19.6 MB/min, Section 5.3), linear extrapolation indicates that LIO-SAM would consume approximately 1176 MB after one hour; this renders extended operation impractical on memory-constrained embedded platforms.

As shown in Figure 1, existing LIO systems exhibit linear (O(N)) memory growth owing to keyframe accumulation. DLIO crashed because of OOM after 6.5 min, and FAST-LIO2 diverged after 25.4 min, and even the relatively efficient LIO-SAM grew at approximately 20 MB/min, limiting extended operation. Detailed measurements are provided in Section 5.

To address this limitation, we propose DB-LIO, a database-driven LiDAR-inertial odometry system. In practice, local map construction, loop closure detection, and graph optimization each use only a subset of the total keyframes. Therefore, DB-LIO persistently stores all keyframes in SQLite with R-Tree spatial indexing while maintaining only the currently required *active window* in memory.

Unlike prior memory management strategies—such as submap freezing in Cartographer [6], working memory/long-term memory (WM/LTM) transfer in RTAB-Map [7], and geometric compression in SLIM [8]—DB-LIO introduces a fundamentally different design principle: *spatially aware cache management tightly coupled with factor-graph optimization*. In existing approaches, memory eviction decisions are made independently of the SLAM backend’s geometric requirements; a recently added but spatially distant node may remain in memory while a nearby older node critical for loop closure is evicted. DB-LIO aligns its cache eviction policy with the spatial requirements of pose-graph optimization by matching the active window radius with the loop closure search radius, guaranteeing that every keyframe potentially involved in the next optimization cycle resides in cache. This tight coupling between spatial indexing and pose-graph optimization is the key methodological distinction that prevents accuracy degradation under memory-bounded operation.

Building on this design principle, the main contributions of this work are summarized as follows:

1. **Spatially Indexed Keyframe Management with Optimization-Aware Caching**: We propose a keyframe management scheme that uses SQLite with R-Tree spatial indexing to persistently store keyframes on disk while supporting efficient spatial queries with O(logN+k) complexity. The key methodological contribution is the tight coupling between the spatial cache eviction policy and the factor-graph optimization requirements: the active window radius is aligned with the loop closure search radius, ensuring that all keyframes relevant to the next graph optimization cycle are guaranteed to reside in cache. This optimization-aware caching distinguishes DB-LIO from prior memory management approaches that treat eviction and optimization independently.
2. **Four-Level Memory Bounding Architecture**: A hierarchical architecture that bounds memory growth to O(C) through (i) SLAM-engine keyframe trimming with transparent on-demand reloading, (ii) a DB-level least recently used (LRU) cache with spatial active window, (iii) Scan Context descriptor pool bounding, and (iv) incremental smoothing and mapping 2 (iSAM2) sliding window compaction with a sparse global anchor graph. Each level targets a distinct memory consumer, and together they enforce a constant memory ceiling for the dominant data structures.
3. **Persistent Storage and Localization Mode**: Unlike conventional SLAM systems that retain only raw map data, the proposed DB-based backend preserves full point clouds and structured metadata—including 6-DoF poses, timestamps, spatial indices, and inter-keyframe relationships—in a queryable database. This enables a dedicated localization mode that can reload previously built maps and perform pose estimation without rebuilding the map. Upon restart, the system performs initial pose estimation via R-Tree spatial queries against the stored map, then switches to real-time tracking through active window-based LIO. The stored map can also be converted to a costmap for immediate navigation without remapping, which is particularly beneficial for agricultural robots that repeatedly operate in the same orchard or field environment.

The remainder of this paper is organized as follows. Section 2 reviews related work, Section 3 and Section 4 describe the system architecture and core algorithms, Section 5 presents experimental results, Section 6 discusses limitations and design trade-offs, and Section 7 concludes the paper.

## 2. Related Work

### 2.1. LiDAR Odometry and SLAM

Recent surveys [9,10] have comprehensively reviewed advances in LiDAR SLAM and identified unbounded memory growth as a key open challenge for extended autonomy. LiDAR odometry and mapping (LOAM) [1] establishes the foundation of LiDAR SLAM by introducing a two-stage approach separating high-frequency odometry from low-frequency mapping. Lightweight and ground-optimized LOAM (LeGO-LOAM) [11] improves efficiency by introducing ground segmentation tailored to ground vehicles. LIO-SAM [2] introduces tightly coupled LiDAR-inertial odometry with IMU preintegration via Georgia Tech smoothing and mapping (GTSAM) [12] factor graph optimization. However, LIO-SAM accumulates all keyframes in memory for loop closure detection, exhibiting O(N) memory growth as characterized by Equation (2).

FAST-LIO2 [3] uses an incremental KD-Tree (ikd-Tree) to process over 10,000 points per millisecond. The ikd-Tree efficiently manages local maps by dynamically inserting and deleting points, but it must maintain the entire map in memory, as shown in Equation (3):

(3) $$M_{\mathrm{FAST}\text{-}\mathrm{LIO}}=\left|\mathrm{ikd}\text{-}\mathrm{Tree}\right|=O\left(N_{\mathrm{points}}\right)$$

Additionally, it lacks loop closure functionality, which limits its ability to correct cumulative drift. Faster-LIO [13] improves computational efficiency using parallel sparse voxels but has a similar memory structure. Direct LiDAR-inertial odometry (DLIO) [14] improves accuracy through continuous-time motion correction but exhibits rapid memory growth owing to dense map storage. Point-LIO [15] achieves high-bandwidth state estimation but likewise exhibits O(N) memory complexity. iG-LIO [16] introduces an incremental generalized iterative closest point (GICP)-based voxel map for efficient neighbor search, but the voxel map remains entirely in memory. Recently, keep it small and simple–iterative closest point (KISS-ICP) [17] proposes a simple yet robust point-to-point ICP, but does not address the memory management problem.

### 2.2. Large-Scale SLAM and Memory Management

Several recent works have addressed map scalability through geometric compression. Scalable and lightweight LiDAR mapping (SLIM) [8] parameterizes point clouds into lightweight line/plane primitives (∼130 KB/km), and multi-scale SLAM (MS-SLAM) [18] applies sliding-window map sparsification to reduce memory by over 70%. Point-based implicit neural SLAM (PIN-SLAM) [19] uses point-based implicit neural representations for compact map storage with global consistency. These approaches compress the map representation itself, which is complementary to DB-LIO’s strategy. DB-LIO retains full-fidelity point clouds—preserving the ability to perform ICP-based loop closure verification and map reuse for future localization—and manages scalability at the storage-and-indexing layer. In principle, geometric compression techniques could be combined with DB-LIO’s storage architecture for further memory savings.

To address scalability issues, Cartographer [6] introduces a submap-based approach. Completed submaps are frozen, with only their metadata kept in memory, so that memory usage scales with the number of submaps rather than the number of raw scans, as shown in Equation (4):

(4) $$M_{\mathrm{Cartographer}}=S\cdot \left|{\mathrm{submap}}_{\mathrm{meta}}\right|+\left|\mathrm{active}\right|=O\left(S\right)$$

where *S* is the number of submaps, and |active| is the size of the currently active submap. However, its reliance on grid-based submaps rather than raw point clouds makes it difficult to apply directly to 3D LiDAR SLAM.

Real-time appearance-based mapping (RTAB-Map) [7] introduces a working memory (WM)/long-term memory (LTM) architecture to limit memory usage:

(5) $$M_{\mathrm{RTAB}\text{-}\mathrm{Map}}=\left|W\right|\leq W_{\max}=O\left(W_{\max}\right)$$

where W is the working memory, and nodes exceeding the threshold are transferred to long-term memory (disk). However, RTAB-Map primarily focuses on visual SLAM and is optimized for visual-feature-based bag-of-words retrieval. LiDAR point clouds require 3D coordinate-based spatial queries, but RTAB-Map does not provide the necessary spatial indexing (e.g., R-Tree). The proposed DB-LIO utilizes the SQLite R-Tree to support spatial range queries with O(logN+k) complexity for LiDAR keyframes, which is directly used for loop closure candidate search and active window updates. Beyond the difference in spatial indexing, DB-LIO and RTAB-Map differ fundamentally in *how* the memory management layer interacts with the SLAM backend. In RTAB-Map, WM/LTM transfer is triggered by a fixed node-count threshold and is agnostic to the geometric layout of stored data; a recently added but spatially distant node may remain in WM while a nearby older node is evicted. This temporal eviction policy can cause accuracy degradation when the robot revisits a previously mapped area, as the keyframes required for loop closure verification may have been transferred to LTM and require disk retrieval. By contrast, DB-LIO aligns its cache eviction policy with the spatial requirements of factor-graph optimization: the active window radius matches the loop closure search radius, ensuring that every keyframe potentially involved in the next graph optimization cycle is guaranteed to reside in cache. This *optimization-aware* spatial caching is a methodological contribution that distinguishes DB-LIO from all prior memory management approaches in SLAM, which treat eviction decisions and optimization requirements independently. Table 1 summarizes the key methodological differences.

### 2.3. Place Recognition

Place recognition corrects cumulative errors through loop closure detection. LiDAR SLAM typically relies on global descriptors such as Scan Context [20], LiDAR Iris [21], and PointNetVLAD [22]. Scan Context encodes height statistics of point clouds in a polar-grid matrix ($N_{r}\times N_{s}$, where $N_{r}$ and $N_{s}$ denote the number of radial rings and azimuthal sectors, respectively), enabling place recognition that is robust to azimuth changes via column-wise circular shifting. Its computational cost is $O(N_{s})$ per candidate comparison, making it suitable for real-time operation.

Learning-based methods such as PointNetVLAD [22] and OverlapNet [23] achieve higher recall in large-scale environments but require graphics processing unit (GPU) inference and large training datasets, making them impractical on embedded platforms such as the Jetson AGX Orin. DB-LIO adopts Scan Context for its lightweight computation and independence from training data, while bounding the descriptor pool size to $S_{\max}$ entries (Section 4.1.6) to prevent the O(N) memory growth that would otherwise arise from storing one descriptor per keyframe.

### 2.4. Database-Based Robotics

In robotics, databases have primarily been used for cloud-based collaboration [24] and spatial data management [25]. Hughes et al. [26] propose hierarchical spatial representations for real-time scene understanding, demonstrating the value of structured spatial indexing in robotics. However, direct integration of spatial databases into real-time SLAM engines remains scarce. SQLite [27] is suitable for embedded environments with its serverless architecture and supports spatial queries with O(logN+k) complexity through its R-Tree extension. Key-value stores such as LevelDB or RocksDB provide high write throughput but do not natively support spatial range queries, making them unsuitable for loop closure candidate search. PostgreSQL/PostGIS provides powerful spatial indexing but requires a server process, introducing excessive overhead for onboard robotic deployments. As a single-file, serverless database with a built-in R-Tree extension, SQLite is well suited for spatially indexed keyframe management in LiDAR SLAM.

### 2.5. Summary of Memory Management in Existing SLAM

The above review reveals two broad regimes among existing 3D LiDAR SLAM systems. LIO-SAM and FAST-LIO2 keep all data in memory and therefore grow as O(N) in the number of keyframes (or $O(N_{\mathrm{pts}})$ in points), posing out-of-memory risks during extended operation. Cartographer and RTAB-Map include memory-limiting mechanisms—submap freezing and WM/LTM hierarchies, respectively—but neither provides spatial indexing for raw LiDAR point clouds, so eviction is decoupled from the geometric requirements of pose-graph optimization. A side-by-side comparison of these complexities together with the empirical numbers measured in this study is given later in Table 3 (Section 5.2), where the theoretical regime predicted here can be checked directly against the observed resident set size (RSS) curves.

## 3. System Architecture

**Figure 2:**
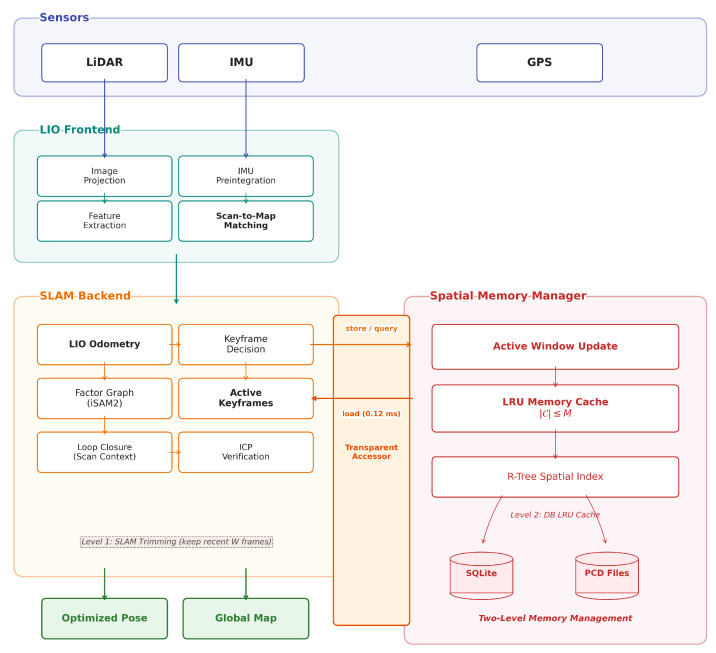
DB-LIO system architecture. The light detection and ranging (LiDAR)-inertial odometry (LIO) frontend (blue) processes LiDAR and inertial measurement unit (IMU) data through feature extraction and scan-to-map matching. The backend (yellow) performs keyframe management, factor graph optimization via incremental smoothing and mapping 2 (iSAM2), and loop closure detection with iterative closest point (ICP) verification. Red arrows indicate data synchronization with the Spatial Memory Manager (pink), which stores keyframe metadata in SQLite and point clouds as point cloud data (PCD) files, enabling spatial queries and on-demand keyframe retrieval.

Figure 2 shows the overall architecture of DB-LIO.

### 3.1. LIO Frontend

The LIO frontend was based on LIO-SAM and performed IMU preintegration [28], point cloud deskewing, and feature extraction to estimate the robot’s relative motion via continuous scan matching.

### 3.2. Graph Optimization and DB Backend

The backend consisted of GTSAM-based factor graph optimization (iSAM2 [29]), loop closure using Scan Context and iterative closest point (ICP), and DB-based keyframe management. The spatial memory manager handled keyframe metadata in SQLite, stored point clouds as point cloud data (PCD) files, answered spatial queries via the R-Tree index, and maintained an LRU memory cache, supplying keyframes for map optimization and loop closure while enforcing memory limits. The SLAM engine’s own keyframe storage was independently trimmed to release point cloud memory beyond the active window; when evicted data were subsequently required, a transparent accessor loaded it from the database on demand, ensuring seamless operation. Place-recognition descriptors were bounded to a fixed pool size ($S_{\max}$), preventing loop closure memory from growing with the number of keyframes. Additionally, the iSAM2 factor graph was periodically compacted via a sliding window mechanism: when the number of poses exceeded a threshold, the Bayes tree was rebuilt with only the most-recent poses, while older poses were preserved in a sparse global anchor graph for inter-window loop closure correction.

## 4. Methodology

### 4.1. Spatial Memory Manager

The spatial memory manager was the core component enabling bounded memory operation.

#### 4.1.1. Database Schema Design

Two SQLite tables stored keyframe information. The **keyframes table** recorded each keyframe’s unique identifier, timestamp, 6-DoF pose (x,y,z,ϕ,θ,ψ) (where ϕ, θ, and ψ denote roll, pitch, and yaw, respectively), PCD file path, and point count. The **R-Tree virtual table** indexed each keyframe by its 3-D axis-aligned bounding box $\left(x_{\min},x_{\max},y_{\min},y_{\max},z_{\min},z_{\max}\right)$, constructed by expanding the keyframe position p=(x,y,z) by ±δ/2 (δ=1.0 m) along each axis. This enabled spatial range queries with O(logN+k) complexity via the SQLite R-Tree extension [30].

#### 4.1.2. Spatial Query Algorithm

Algorithm 1 describes the spatial query process for retrieving keyframes within a specified radius.
**Algorithm 1** Spatial Keyframe Query via R-Tree**Require:** 
Current pose $p_{c}=\left(x_{c},y_{c},z_{c}\right)$, search radius *r*, max count *k***Ensure:** 
Keyframe ID list I sorted by distance  1:$B\leftarrow [x_{c}\pm r,\;y_{c}\pm r,\;z_{c}\pm r]$         ▹ Query bounding box  2:$I_{\mathrm{raw}}\leftarrow \mathrm{RTreeRangeQuery}\left(T_{\mathrm{rtree}},\;B\right)$  3:$I\leftarrow \mathrm{SortByDistance}(I_{\mathrm{raw}},\;p_{c})$  4:I←I[1:k]             ▹ Retain at most *k* nearest  5:**return** I

#### 4.1.3. Memory Cache Management

The cache maintained up to *M* keyframes in memory, as shown in Equation (6):

(6) $$\left|C\right|\leq M,\;C=\{\left(id_{i},P_{i}\right)|id_{i}\in A\}$$

where A is the set of active keyframe IDs determined by spatial proximity to the current pose. When the cache exceeded its limit *M*, entries not in the current active set A were evicted in LRU order until |C|≤M.

#### 4.1.4. Active Window Update

As the robot moved, the active window was updated by issuing a spatial query (Algorithm 1) centered at the current pose with radius $r_{q}$. Algorithm 2 formalizes the active window update process, which manages the transition between old and new active sets and triggers cache eviction.
**Algorithm 2** Active Window Update**Require:** 
Current pose $p_{c}$, active window radius $r_{q}$, cache limit *M*, previous active set $A_{\mathrm{old}}$**Ensure:** 
Updated active set $A_{\mathrm{new}}$, updated cache C  1:$A_{\mathrm{new}}\leftarrow \mathrm{SpatialQuery}\left(p_{c},r_{q}\right)$                      ▹ Algorithm 1  2:$A_{\mathrm{load}}\leftarrow A_{\mathrm{new}}\setminus A_{\mathrm{old}}$                   ▹ Newly entering keyframes  3:$A_{\mathrm{evict}}\leftarrow A_{\mathrm{old}}\setminus A_{\mathrm{new}}$                       ▹ Departing keyframes  4:**for** $id\in A_{\mathrm{load}}$ **do**                   ▹ Preload new keyframes into cache  5:    **if** id∉C **then**  6:        C[id]←D.loadCloud(id)  7:    **end if**  8:**end for**  9:**for** $id\in A_{\mathrm{evict}}$ **do**            ▹ Mark departed keyframes as eviction candidates  10:    C.markEvictable(id)  11:**end for**  12:**while** |C|>M**do**                      ▹ Enforce cache limit via LRU  13:    $id_{\mathrm{lru}}\leftarrow C.\mathrm{getLRU}\left(\mathrm{evictable}\;\mathrm{only}\right)$  14:    $C.\mathrm{remove}(id_{\mathrm{lru}})$  15:**end while**  16:$A_{\mathrm{old}}\leftarrow A_{\mathrm{new}}$  17:**return** $A_{\mathrm{new}},C$

The critical design choice was that $r_{q}$ matched the loop closure search radius $r_{lc}$, ensuring that every keyframe potentially involved in the next graph optimization cycle is guaranteed to reside in cache. Keyframes in $A_{\mathrm{old}}\setminus A_{\mathrm{new}}$ became eviction candidates, and the memory limit was enforced after each update via LRU ordering.

#### 4.1.5. Two-Level Memory Management

Critically, bounding only the DB-level LRU cache was insufficient: the SLAM engine’s internal keyframe storage retained references to every point cloud ever created, which prevented actual memory release even when the DB cache evicted entries.

To address this, DB-LIO introduced a *two-level* memory management architecture as follows.

**Level 1—SLAM-Engine Trimming.** The SLAM engine periodically released point cloud data for keyframes older than the most-recent *W* entries, where *W* is the active keyframe window size (default 100). The storage structure size was preserved to maintain index consistency with the pose graph; however, the actual point cloud memory was released, as shown in Equation (7):

(7) $$V\left[i\right]\leftarrow \left\{\begin{array}{cc} P_{i} & \mathrm{if}\;i\geq N-W\\ ⌀ & \mathrm{otherwise} \end{array}\right.$$

**Level 2—DB-Level LRU Cache.** The spatial memory manager’s LRU cache, Equation (6), served as a secondary cache that independently managed disk-loaded point clouds.

**Transparent Accessor.** A unified access interface was provided for all keyframe point cloud retrieval, managing synchronization between the SLAM-engine’s trimmed storage (Level 1) and the DB cache (Level 2). When a downstream consumer requested keyframe *i*, the accessor first checked whether Level 1 (the SLAM engine’s internal vector V) held the point cloud. If V[i] had been trimmed (set to ⌀), the accessor fell through to Level 2 (the DB cache C). If the keyframe existed in C, it was returned directly without a disk read; otherwise, the accessor loaded it from the SQLite database and inserted it into C, triggering LRU eviction if the cache limit was exceeded. This two-level lookup prevented redundant disk loads: a keyframe still held in the SLAM engine’s active window was never re-fetched from disk, while a trimmed keyframe already cached by a prior spatial query was served from the cache. All downstream consumers (local map construction, loop closure verification, visualization) operated identically regardless of whether data resided in memory or on disk. Algorithm 3 formalizes this process.
**Algorithm 3** Transparent Keyframe Access**Require:** 
Keyframe index *i*, vector V, spatial memory manager D**Ensure:** 
Point cloud $P_{i}$ or null  1:**if** V[i]≠null **then**  2:     **return** V[i]                   ▹ Level-1 hit  3:**else if** DB mode enabled **then**  4:     $P_{i}\leftarrow D$.loadCloud(*i*)             ▹ Level-2 / disk load  5:     **return** $P_{i}$  6:**else**  7:     **return** null  8:**end if**

#### 4.1.6. Scan Context Descriptor Bounding

Place-recognition descriptors also contributed to O(N) memory growth. Scan Context [20] stored a polar-grid descriptor matrix ($N_{r}\times N_{s}$) per keyframe, along with ring keys, sector keys, and a KD-tree for fast retrieval. Without explicit management, descriptor memory increased linearly with the number of keyframes.

DB-LIO bounded the Scan Context descriptor pool to a maximum of $S_{\max}$ entries (default 2000). When the pool exceeded $S_{\max}$, the oldest $S_{\max}/2$ entries were removed, and the KD-tree was rebuilt over the remaining descriptors, as shown in Equation (8):

(8) $$\left|S\right|>S_{\max}\Rightarrow \left\{\begin{array}{c} S\leftarrow S[S_{\max}/2:]\\ \Delta_{\mathrm{offset}}+=S_{\max}/2 \end{array}\right.$$

A global index offset $\Delta_{\mathrm{offset}}$ was maintained so that the returned loop candidate IDs remained consistent with the pose graph’s keyframe indices: global_id = local_id + Δ_offset_. This ensured that loop closure operated correctly even after multiple trimming cycles, while the descriptor memory was bounded at $O(S_{\max})$.

#### 4.1.7. iSAM2 Sliding Window Compaction

Standard iSAM2 maintained a Bayes tree over all pose variables, whose memory grew as O(N) with the number of keyframes *N*. Sliding window and marginalization techniques have been explored to limit factor graph size [31], but applying them to LiDAR-inertial SLAM with bounded memory guarantees remains an open problem. DB-LIO introduced a two-level factor graph architecture to bound this growth as follows.

**Level 1—Local iSAM2 Window.** A sliding window of at most $W_{\max}$ poses (default 500) was maintained in the active iSAM2 instance. When the window exceeded $W_{\max}$, the system performed *compaction*: a new iSAM2 instance was created with only the most-recent $W_{\mathrm{keep}}$ poses (default 200), and the old instance was discarded. A prior factor anchored the oldest-retained pose to preserve absolute consistency, as shown in Equation (9).

(9) $$W_{\mathrm{active}}\leq W_{\max},\;\mathrm{after}\;\mathrm{compaction}:\;W_{\mathrm{active}}=W_{\mathrm{keep}}$$

**Level 2—Sparse Global Anchor Graph.** For every $A_{\mathrm{int}}$ keyframes (default 100, the anchor registration interval), an *anchor* keyframe was registered in a sparse global factor graph. Loop closures involving poses outside the local window were mapped to their nearest anchors and added as between-factors in the global graph. When a loop closure triggered global optimization, the anchor graph was solved, and the resulting corrections were propagated to all keyframe poses, as shown in Equation (10).

(10) $$G_{\mathrm{global}}=\left\{\left(T_{a_{i}},T_{a_{j}},\Delta_{ij}\right)|a_{i},a_{j}\in N_{\mathrm{anc}}\right\}$$

where $N_{\mathrm{anc}}$ is the set of anchor keyframe indices (distinct from the active set A defined in Section 4.1) and $\Delta_{ij}$ is the relative pose constraint between anchors $a_{i}$ and $a_{j}$. This two-level design bounded the iSAM2 Bayes tree at $O(W_{\mathrm{keep}})$ memory while preserving global consistency through the sparse anchor graph.

**Supplementary Memory Optimizations.** In addition to the four main bounding levels, several auxiliary techniques reduced memory fragmentation and prevented unbounded growth of secondary data structures: (i) the visualization path buffer was capped at 5000 poses, (ii) the local map cache was cleared when exceeding 300 entries, and (iii) explicit heap defragmentation was performed after loop closure pose corrections, periodically after every 100 keyframes, to return freed memory to the operating system, mitigating allocator fragmentation that otherwise prevented resident memory reduction even after deallocation.

### 4.2. Loop Closure Module

The loop closure module was decoupled from the map optimization module for modularity and operated in dual mode:

- **Memory Mode**: All keyframes resided in an in-memory container, and candidates were searched through direct index access.
- **DB Mode**: Candidates were found via spatial queries (Algorithm 1), and the required point clouds were retrieved from the LRU cache or loaded from the disk.

Loop closure proceeded in three stages: candidate selection, geometric verification, and factor addition. During candidate selection, a radius-based search identified keyframes within $r_{lc}$ of the current position whose index gap from the current keyframe exceeded $\Delta_{\mathrm{idx}}$ (default 30, to exclude temporally adjacent keyframes). The index-based criterion replaced a time-based one used in the original LIO-SAM to avoid false positives when the robot was stationary, as shown in Equation (11):

(11) $$C_{r}=\{i|\;∥p_{i}-p_{c}{∥}_{2}\;<\;r_{lc}\wedge |i-i_{c}\left|\;>\;\Delta_{\mathrm{idx}}\right\}$$

Within DB-LIO, Scan Context descriptors served as the primary place recognition mechanism: candidates identified by the spatial query were matched against the bounded descriptor pool (Section 4.1.6) to detect revisited locations. Selected candidates then underwent ICP registration [32] and had to satisfy convergence, fitness score, and translation/rotation magnitude thresholds to be accepted. Accepted loops were added to the pose graph as between-factors with a robust Cauchy noise model [33] (kernel parameter k=1, diagonal covariance $\sigma^{2}=0.3$ m^2^) to mitigate the effect of occasional false loop closures. For stability, the system tracked per-keyframe failure counts, paused after consecutive failures, and enforced a loop count limit within a sliding window.

**Asynchronous integration and stale-candidate prevention.** Although the loop closure thread runs asynchronously from the main optimization thread, stale candidates—i.e., loops detected against outdated states or duplicates of already-integrated pairs—are filtered through a producer–consumer pipeline: an index-gap rejection ($|k_{\mathrm{cur}}-k_{\mathrm{pre}}|\;<\;\Delta_{\mathrm{idx}}$), a per-pair duplicate-suppression hash, a mutex-protected single-writer queue, drain-and-clear semantics that bound staleness to one optimization cycle, and a compaction-aware skip that excludes factors referencing frozen poses. Together, these guarantees ensure that every loop factor reaching the graph is unique, at most one cycle old, and references only currently optimizable poses.

### 4.3. Persistent Storage and Localization Mode

Existing LIO frameworks relied on in-memory structures, so all map data were lost when the system shut down. DB-LIO utilized SQLite-based persistent storage to preserve all keyframes on disk, supporting dual-operation modes:

- **Mapping Mode**: Explored new environments, built a map, and saved it to the DB.
- **Localization Mode**: Loaded a map from an existing DB and performed pose estimation only.

In localization mode, the system loaded a stored map and estimated the initial pose via R-Tree spatial queries, then switched to real-time tracking through active window-based LIO. Upon restart, the stored map could be converted to a costmap for immediate navigation without remapping. This significantly reduced remapping overheads for logistics robots, agricultural robots, and other applications that operated repeatedly in the same environment.

### 4.4. Theoretical Analysis of O(C) Memory Convergence

We analyzed how the memory usage of DB-LIO converged to a bounded level regardless of map size. The total memory of the system consisted of four bounded components:

**(1) SLAM-engine keyframe vector.** The SLAM-engine trimming mechanism ensured that at most *W* entries retained actual point cloud data, Equation (7), contributing $W\cdot m_{k}$ memory, where $m_{k}$ is the average memory per keyframe.

**(2) DB-level LRU cache.** The spatial memory manager cache held at most $C_{\mathrm{db}}$ entries, Equation (6), contributing $C_{\mathrm{db}}\cdot m_{k}$ memory. In practice, $W=C_{\mathrm{db}}$ (both default to 100), but they could be configured independently.

**(3) Scan Context descriptor pool.** The descriptor pool was bounded at $S_{\max}$ entries, Equation (8), each consuming approximately 4.8 KB (20×60 float matrix plus keys), contributing $S_{\max}\cdot m_{s}$ memory.

**(4) iSAM2 Bayes tree.** The sliding-window compaction (Section 4.1.7) bounded the Bayes tree to at most $W_{\mathrm{keep}}$ pose variables (default 200), contributing $W_{\mathrm{keep}}\cdot m_{g}$ memory, where $m_{g}$ is the average memory per pose node in the Bayes tree. The sparse global anchor graph grew as $O(N/A_{\mathrm{int}})$ with anchor interval $A_{\mathrm{int}}$. However, each anchor stored only a single SE(3) pose (48 bytes). Therefore, its contribution was negligible compared with that of the point cloud memory.

Therefore, the total memory was given by Equation (12):

(12) $$\begin{array}{cc} M\left(t\right)=M_{0} & +\min\left(N\left(t\right),W\right)\cdot m_{k}+\min\left(N\left(t\right),C_{\mathrm{db}}\right)\cdot m_{k}\\ \; & +\min\left(N\left(t\right),S_{\max}\right)\cdot m_{s}+W_{\mathrm{keep}}\cdot m_{g} \end{array}$$

where $M_{0}$ is the base process memory and N(t) is the keyframe count up to time *t*.

After cache saturation ($N\left(t\right)>\max\left(W,C_{\mathrm{db}},S_{\max},W_{\mathrm{keep}}\right)$), Equation (12) changed to Equation (13):

(13) $$\begin{array}{cc} M(t) & \approx M_{0}+W\cdot m_{k}+C_{\mathrm{db}}\cdot m_{k}\\ \; & \;+S_{\max}\cdot m_{s}+W_{\mathrm{keep}}\cdot m_{g}=O\left(C\right) \end{array}$$

where $C=W+C_{\mathrm{db}}+S_{\max}+W_{\mathrm{keep}}$ represents the total bounded capacity. The total memory was decomposed as:

(14) $$M_{\mathrm{total}}\left(t\right)=\underset{\mathrm{dense}\;\mathrm{data}}{\underset{︸}{O(C)}}+\underset{\mathrm{metadata}}{\underset{︸}{O(N_{\mathrm{sparse}})}}$$

where the O(C) term covers the four bounded components (hundreds of MB) and $O(N_{\mathrm{sparse}})$ covers ancillary structures (anchor graph at 48 bytes/entry, loop history) that grow at a negligible rate. The observed residual growth (7.7 MB/min post-saturation) was primarily attributable to heap fragmentation rather than architectural leakage (Section 6). Without these bounds—i.e., in the *DB OFF* configuration, in which the four bounding levels and DB-based keyframe offloading are disabled while the rest of the DB-LIO pipeline (loop closure, Scan Context, and robust noise modeling) is preserved (formal definition in Section 5.1)—all four components grew with *N*, yielding $M\left(t\right)=M_{0}+N\left(t\right)\cdot m_{k}=O\left(N\right)$. Even for the lighter LIO-SAM baseline (19.6 MB/min from Table 4), memory would reach approximately 1176 MB after 1 hour, posing OOM risks on memory-constrained embedded platforms. Experimental validation of this convergence is presented in Section 5.3.

#### Optimization-Aware Cache Guarantee

Beyond bounding memory, the spatial caching design provides a formal guarantee that memory reduction does not degrade optimization quality.

**Theorem** **1** (Cache Hit Guarantee)**.** *Let $p_{i}$ denote the current robot pose, $r_{lc}$ the loop closure search radius, $r_{q}$ the active window query radius, and $C_{\mathrm{db}}$ the LRU cache capacity. Define the local keyframe density at time t as $\rho \left(t,r_{q}\right)=|\{k_{j}:\;∥p_{i}\left(t\right)-p_{j}∥\;\leq \;r_{q}\}|$. If*

(15) $$r_{q}\geq r_{lc}\;\mathrm{and}\;\rho \left(t,r_{q}\right)\leq C_{\mathrm{db}},\;\forall \;t,$$

*then for every keyframe $k_{j}$ satisfying the loop closure candidate criterion $∥p_{i}\left(t\right)-p_{j}∥\;\leq \;r_{lc}$, $k_{j}$ is guaranteed to reside in the LRU cache at time t. That is, the cache miss rate for optimization-relevant keyframes is zero.*

**Proof.**  At each active window update (Algorithm 2), the system performs an R-Tree spatial query $Q\left(p_{i},r_{q}\right)=\{k_{j}:\;∥p_{i}-p_{j}∥\;\leq \;r_{q}\}$ and loads all results into the cache. Since $r_{q}\geq r_{lc}$, the loop closure candidate set satisfies $\{k_{j}:\;∥p_{i}-p_{j}∥\;\leq \;r_{lc}\}\subseteq Q\left(p_{i},r_{q}\right)$, so all candidates are loaded. The condition $\rho \left(t,r_{q}\right)\leq C_{\mathrm{db}}$ ensures that the loaded set fits entirely within the cache without triggering LRU eviction of any spatially relevant keyframe.    □

**Corollary** **1** (Optimization Equivalence)**.** *Under the conditions of Theorem 1, the set of keyframes available for loop closure detection and factor-graph optimization at each time step is identical to that of an unbounded system retaining all keyframes in memory. Consequently, the optimization result—and thus the trajectory accuracy—is independent of the memory bounding mechanism.*

Condition (15) relates the cache capacity $C_{\mathrm{db}}$ to the environment’s spatial keyframe density ρ. The ablation study (Table 7) confirms a cache hit rate of 90.8% at the default C=100, indicating that the density condition was satisfied for the vast majority of optimization events. Even at C=25, the hit rate remained at 65.5%, and on-demand disk loads (0.12 ms average) provided graceful degradation without affecting real-time odometry.

**Remark** **1** (Behavior under condition violation)**.** *The density condition $\rho \left(t,r_{q}\right)\leq C_{\mathrm{db}}$ may be violated when the robot revisits a densely mapped area whose spatial query returns more than $C_{\mathrm{db}}$ keyframes within $r_{q}$. In this regime, the LRU policy evicts the least recently used keyframes within the active window; DB-LIO handles the resulting cache misses via two complementary mechanisms—on-demand SQLite reads as a fallback (so no loop-closure candidate is silently dropped) and the per-step re-prioritization in Algorithm 2 that realigns the cache within one update cycle once the dense area is left. The cache-size ablation (Table 7) bounds the practical cost: at the smallest tested C=25, the hit rate dropped to 65.5% and the ATE rose modestly from the default 0.305 m at C=100 to 0.408 m, confirming that the system remains operational under partial violation albeit with a measurable accuracy cost. Sustained pathological cases ($\rho \gg C_{\mathrm{db}}$, e.g., very dense indoor environments) require enlarging $C_{\mathrm{db}}$ or shrinking $r_{q}$; Section 5.5 provides design guidance for this trade-off.*

## 5. Experimental Results and Performance Comparison

This section presents experiments that validate the proposed DB-based SLAM system. We evaluated memory efficiency, trajectory accuracy, and localization mode practicality on a custom orchard dataset and the Karlsruhe Institute of Technology and Toyota Technological Institute at Chicago (KITTI) odometry benchmark [34]. Together, these datasets span two LiDAR sensor types and distinct environments, demonstrating the generalizability of the system.

### 5.1. Experimental Setup

This subsection describes the hardware platform, sensor suite, test environments, and comparison targets used in our experiments. We evaluated DB-LIO on two complementary datasets: a custom orchard dataset collected with a Livox Mid360 LiDAR to test memory bounding under extended operation, and the public KITTI odometry benchmark [34] with a Velodyne HDL-64E to validate generalization across different LiDAR types and environments.

#### 5.1.1. Platform, Sensors, and Test Environment

The mobile platform was an AgileX Scout Mini (AgileX Robotics, Shenzhen, China), a four-wheeled skid-steer robot (Figure 4b). The sensor suite comprised a Livox Mid360 LiDAR (Livox Technology Co., Ltd., Shenzhen, China) (nonrepetitive scanning, 200 m range), its built-in IMU (200 Hz), and a TDR3000 real-time kinematic global navigation satellite system (RTK-GNSS) receiver providing centimeter-level global positioning system (GPS) ground truth. The RTK receiver maintained a fixed solution (Fix status) throughout all data collection runs, with a nominal horizontal accuracy of ±2 cm under open-sky conditions; although canopy occlusion in the orchard could intermittently degrade this to ±5–10 cm, the overall accuracy remained sufficient for evaluating meter-level SLAM trajectories. All sensor data were recorded as robot operating system (ROS) 2 [35] rosbags for offline reproducibility.

**Figure 3:**
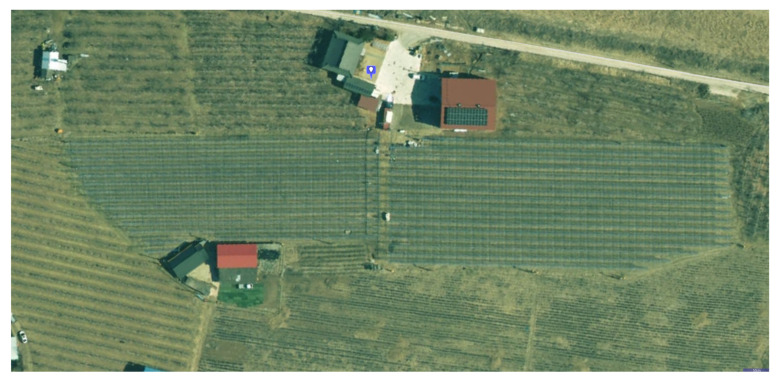
Satellite view of the apple orchard test site in Andong, South Korea.

**Figure 4:**
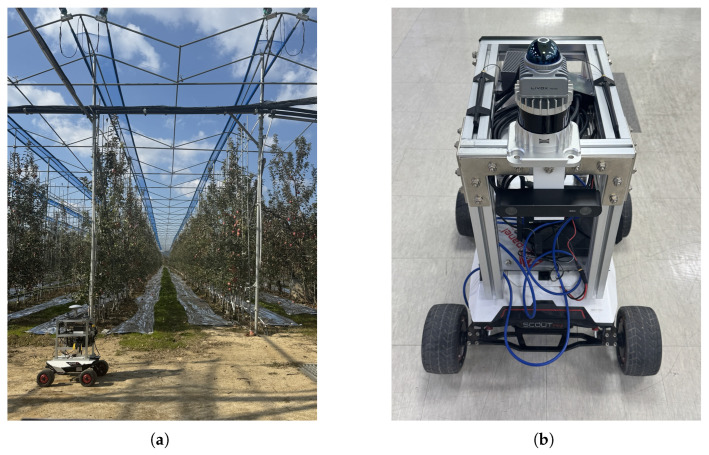
(**a**) Ground-level view of the orchard corridor (∼2 m wide) with the Scout Mini robot. (**b**) Sensor platform: AgileX Scout Mini with Livox Mid360 LiDAR and TDR3000 RTK-GNSS.

As shown in Figure 3 and Figure 4, the custom dataset was collected in a structured apple orchard in Andong, Republic of Korea. The orchard comprised regularly spaced tree rows separated by narrow corridors (approximately 2 m wide), forming an elongated corridor-like environment in which the robot traversed back and forth along the rows. This layout posed specific challenges for SLAM: (i) highly repetitive visual and geometric features across adjacent rows increased the risk of perceptual aliasing in loop closure, and (ii) the elongated, narrow geometry limited lateral point cloud overlap, making scan matching more sensitive to longitudinal drift. The dataset comprised 37.5 min of continuous operation and covered the full orchard area.

**Keyframe insertion characteristics.** Throughout the 37.5 min run, DB-LIO inserted 5867 keyframes (≈156.4 per min, ≈2.6 Hz; Table 6); the 90.8% cache hit rate at $r_{q}\;=\;50$ m and $C_{\mathrm{db}}\;=\;100$ empirically confirms that the density condition $\rho \left(t,r_{q}\right)\;\leq \;C_{\mathrm{db}}$ assumed in Theorem 1 held throughout.

#### 5.1.2. Public Benchmark Dataset

In addition to the custom dataset, the KITTI odometry benchmark [34] was used. KITTI is an urban driving dataset collected with a Velodyne HDL-64E (64-channel mechanical LiDAR). Sequences 00, 05, and 07 were used for the localization mode validation. Together with the custom orchard dataset (Livox Mid360), these covered two different LiDAR sensor types and distinct environment types (orchard, urban driving).

#### 5.1.3. Comparison Targets and Computing Environment

Experiments were conducted on two platforms serving complementary roles. The **desktop PC** (Intel i7-12700, 32 GB DDR5, Samsung 990 PRO NVMe SSD) was used for the head-to-head baseline comparison, ensuring that all systems (DB-LIO and baselines) ran under identical hardware. The **target deployment platform** was an NVIDIA Jetson AGX Orin (ARM Cortex-A78AE, 32 GB LPDDR5, NVMe SSD), used for on-robot data collection and for validating the full four-level memory bounding architecture (Section 5.9). Baselines were LIO-SAM, Point-LIO, DLIO, and FAST-LIO2, together with RTAB-Map [7] as a bounded-memory reference.

**Bounding-level configuration and platform mapping.** The desktop PC was used for the head-to-head DB-LIO experiments (Section 5.2, Section 5.3, Section 5.4, Section 5.5 and Section 5.6) and the KITTI localization run (Section 5.8); the DB-LIO row in Table 4 and all baselines other than RTAB-Map are these desktop measurements, and they activated three of the four bounding levels (keyframe trimming, LRU cache, descriptor pool bounding). The Jetson AGX Orin was used for two on-robot experiments: the bounded-memory comparison with RTAB-Map (Section 5.7, also at three levels for fair comparison; the RTAB-Map row in Table 4 is this Jetson measurement) and the full four-level validation that additionally enabled iSAM2 sliding window compaction (Section 5.9). The fourth level was deferred to the Jetson run because its effect is most visible under the larger Bayes tree that accumulates over long on-robot operation; consequently, the desktop results represent a conservative (slightly higher) upper bound on the expected four-level footprint.

**DB OFF semantics.** Several comparisons below include a “DB-LIO (DB OFF)” configuration. This is *not* equivalent to vanilla LIO-SAM: it runs the full DB-LIO codebase (including the enhanced loop closure pipeline with Scan Context and robust noise modeling), but with all four bounding levels and DB-based keyframe offloading disabled, so that keyframes, descriptors, and graph variables accumulate in memory without eviction. DB OFF isolates the effect of the bounding mechanism from the effect of the loop closure pipeline.

All experiments were repeated at least twice under identical conditions; reported values represented means across runs, with standard deviations below 1% for all trajectory metrics (e.g., absolute trajectory error (ATE) RMSE 0.305 ± 0.001 m), confirming high reproducibility. Table 2 summarizes the key implementation parameters.

### 5.2. Memory Footprint and Stability

Before presenting the empirical results, Table 3 restates the theoretical memory complexities of the systems under comparison.

**Figure 5:**
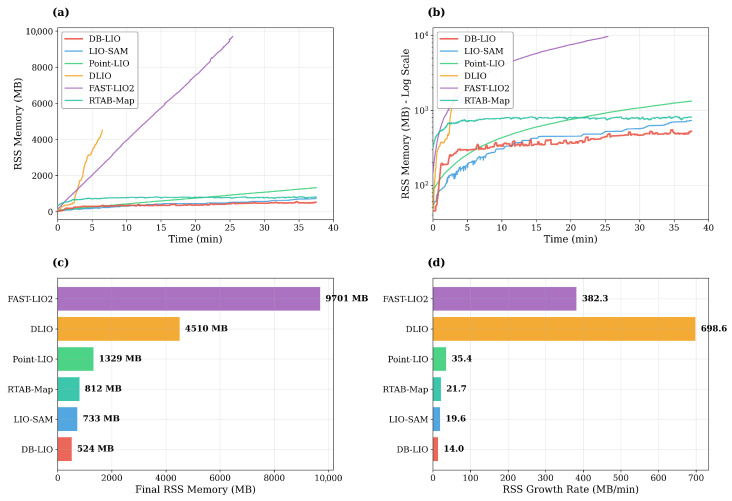
Memory usage comparison of six SLAM systems over 37.5 min. (**a**) RSS memory over time (linear scale). (**b**) RSS memory over time (log scale). (**c**) Final RSS memory at the end of each run. (**d**) Average RSS growth rate. DB-LIO maintained a bounded memory level after reaching the LRU cache limit, in contrast to the O(N) growth shown in Figure 1.

The theoretical complexities in Table 3 predicted that systems with O(N) growth would eventually exhaust available memory, while DB-LIO’s O(C) design would remain bounded. The experimental results confirmed this prediction. As shown in Figure 5 and Table 4, during 37.5 min of mapping, the memory usage of LIO-SAM grew linearly (O(N)) to 733 MB at a rate of 19.6 MB/min, while DB-LIO reached only 524 MB (14.0 MB/min overall). As a cross-platform reference for bounded-memory SLAM, RTAB-Map [7]—which also employs bounded memory management and was executed on the Jetson AGX Orin (its native deployment target; see Section 5.7 for the rationale)—reached 812 MB final RSS (21.7 MB/min overall). The key difference lay in the eviction policy and its downstream effect on SLAM quality. After cache saturation (from approximately 3 min onward), the growth rate of DB-LIO dropped to 7.7 MB/min, 61% lower than that of LIO-SAM. DLIO crashed because of memory exhaustion after 6.5 min, and FAST-LIO2 diverged after 25.4 min.

### 5.3. DB Mode ON/OFF Comparison

To isolate the effect of the DB module, we ran experiments with the DB mode enabled and disabled under identical conditions on the same dataset, using the DB OFF configuration defined in Section 5.1 (DB OFF semantics): that is, the full DB-LIO codebase with all four bounding levels and DB-based keyframe offloading disabled. The OFF baseline reached 2888 MB, which exceeded the footprint of LIO-SAM (733 MB, Table 4) because the richer loop closure pipeline of DB-LIO retained additional per-keyframe data structures. Table 5 compares both memory usage and trajectory accuracy after 37.5 min of operation.

Activating the DB mode significantly reduced memory because the LRU cache policy offloaded inactive keyframes to the disk, Scan Context descriptor pool bounding limited the descriptor memory, and periodic heap defragmentation (malloc_trim) reclaimed the freed memory. After cache saturation (from approximately 3 min onward), the growth rate of DB-LIO dropped to 7.7 MB/min, demonstrating bounded-rate memory behavior. The memory curve exhibited a characteristic sawtooth pattern: memory rose gradually as new keyframes were created, and dropped when the LRU cache evicted inactive keyframes and the allocator reclaimed the freed heap. This confirmed that the first three bounding levels (keyframe trimming, LRU cache, descriptor pool) operated as designed. The post-saturation growth rate was 90% lower than that of the DB OFF baseline, which was 78.4 MB/min, confirming effective bounded-rate convergence. As shown in Table 5, the DB mode ON achieved an 81.9% reduction in the final RSS while maintaining a comparable trajectory accuracy (ATE RMSE 0.305 m vs. 0.296 m). Compared with vanilla LIO-SAM (733 MB, Table 4), DB-LIO also reduced memory by 28.5% without degrading trajectory accuracy.

**Figure 6:**
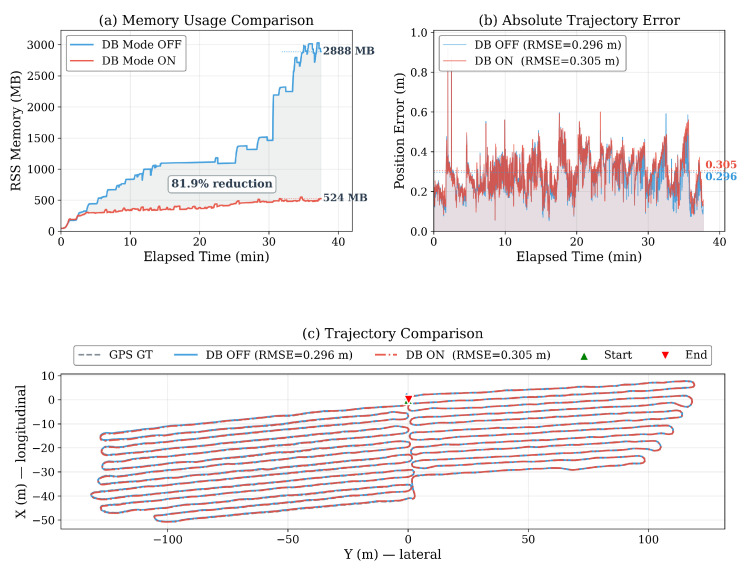
DB mode ON/OFF comparison. (**a**) Memory usage over time: the DB mode ON bounded RSS memory via LRU eviction, achieving 81.9% reduction. (**b**) Absolute trajectory error over time: both modes exhibited comparable accuracy. (**c**) Trajectory comparison against GPS ground truth (axes follow the ROS body-frame convention: X = longitudinal, Y = lateral).

Figure 6 presents a comprehensive comparison. Panel (a) shows that memory grows linearly with the DB mode OFF, while the DB mode ON maintained a bounded level after reaching the LRU cache limit, with periodic drops caused by LRU cache eviction and heap reclamation. Panel (b) confirms that the ATE remains comparable between the two modes throughout the entire trajectory. Panel (c) overlays the estimated trajectories, demonstrating that both modes closely follow the GPS ground truth.

### 5.4. I/O Overhead of DB Mode

The DB mode achieved near-identical performance on cache hits (<0.01 ms) compared with that of memory mode, with only 0.09–0.12 ms average disk load delay on cache misses (NVMe SSD). The active window technique empirically achieved a cache hit rate of 90.8%, keeping most keyframes required for loop closure verification in the cache. Table 6 summarizes the cache performance on the custom orchard dataset.

In the worst case of consecutive cache misses (e.g., re-entering a previously visited area), up to 10 candidates could require disk loading, totaling at most 10×0.12ms≈1.2ms. As the loop closure module operated asynchronously in a separate thread from the main LIO odometry, this delay did not affect real-time pose estimation and was negligible compared with that of the 10 Hz LiDAR frame period (100 ms).

### 5.5. Ablation Study: Cache Size and Active Window Radius

To analyze the impact of the two key hyperparameters—LRU cache size *C* and active window radius $r_{q}$—on memory usage and trajectory accuracy, we conducted an ablation study on the custom orchard dataset.

#### 5.5.1. Effect of Cache Size

Table 7 shows the effect of varying cache size while fixing the active window radius at 50 m.

#### 5.5.2. Effect of Active Window Radius

Table 8 shows the effect of varying the active window radius while fixing the cache size at 100 frames.

The cache size ablation demonstrated a clear trade-off between memory efficiency and localization quality. At C=25, the cache was too small to retain enough keyframes for reliable loop closure verification, resulting in a degraded ATE of 0.408 m with only 65.5% cache hit rate; frequent cache misses forced repeated disk loads, and some loop closure candidates were unavailable in time for ICP verification. At C=50, accuracy improved substantially (ATE 0.326 m, hit rate 83.0%), but the hit rate remained below 90%, indicating occasional missed loop closures. The default C=100 achieved a favorable balance: 90.8% hit rate with ATE 0.305 m, nearly matching the unbounded baseline (0.296 m) at only 18.1% of its memory cost. Increasing to C=200 yielded diminishing returns in accuracy (ATE 0.293 m, only 4% improvement over C=100) while tripling the memory footprint (1754 MB), suggesting over-provisioning for this environment. These results indicated that the optimal cache size depended on the spatial density of loop closure candidates: environments with denser revisitation patterns may benefit from larger caches, while sparse environments could operate effectively with smaller caches.

By contrast, the active window radius showed minimal impact across the tested range (30–100 m). Two properties of the orchard environment account for this saturation: the loop closure search radius is small ($r_{lc}\;=\;10$ m, Table 2), so even at $r_{q}\;=\;30$ m the active window already encloses every loop closure candidate, and the corridor-like layout has near-uniform keyframe density along its longitudinal axis, so the LRU residency profile is nearly invariant across the tested radii. The radius parameter is expected to become significantly more impactful in environments that violate either property—e.g., long-range loop closures with $r_{lc}\;>\;r_{q}$ (where the spatial active-window guarantee in Theorem 1 breaks), spatially heterogeneous environments with order-of-magnitude density variation (urban plazas, multi-floor indoor sites), or high-speed platforms whose keyframes spread sparsely along several hundred meters per minute. These results provide practical parameter-selection guidance: the cache size $C_{\mathrm{db}}$ should be tuned to match the expected spatial density of loop closure candidates, while the active window radius $r_{q}$ should be set to be at least the loop closure search radius $r_{lc}$ (and increased further only when loop-closure operations span larger distances) to preserve the optimization-aware caching guarantee.

### 5.6. Trajectory Accuracy

**Figure 7:**
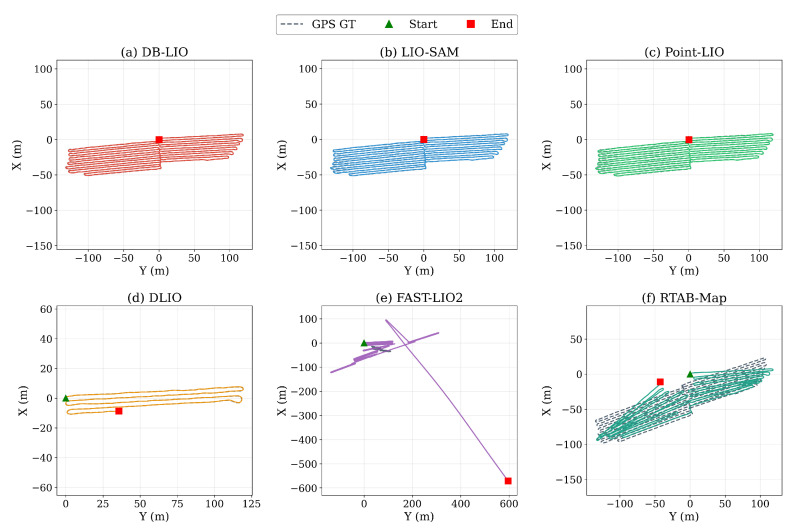
Trajectory comparison on the custom dataset. (**a**) DB-LIO. (**b**) LIO-SAM. (**c**) Point-LIO. (**d**) DLIO (stopped at 6.5 min owing to OOM). (**e**) FAST-LIO2 (diverged after 25.4 min). (**f**) RTAB-Map (LiDAR-only configuration; visual loop closure inactive—analyzed in Section 5.7). GPS ground truth is shown as a dashed line in each panel.

Beyond memory efficiency, it was necessary to verify that DB-LIO did not sacrifice trajectory accuracy. The ATE was measured against GPS ground truth on the custom orchard dataset using the evo evaluation tool [36]. Table 9 and Figure 7 compare the overall trajectory accuracy of DB-LIO against existing LIO systems.

As shown in Table 9 and Figure 7, DB-LIO achieved competitive trajectory accuracy (ATE RMSE 0.305 m). DLIO and FAST-LIO2 were excluded from this comparison because they terminated early owing to OOM or divergence (see Table 4), making their partial-trajectory metrics not directly comparable. Among the systems that completed the full 37.5 min run, DB-LIO achieved lower ATE than that of both LIO-SAM (0.305 m vs. 0.508 m) and Point-LIO (0.516 m). RTAB-Map exhibited substantially higher ATE (RMSE 11.166 m), attributable to the pipeline mismatch (zero visual loop closures in the pure LiDAR setting) rather than a failure of its memory management; this is analyzed in detail in Section 5.7. To clarify the source of this accuracy improvement, Table 9 also included DB-LIO (DB OFF), which ran the same enhanced loop closure pipeline but with all four memory bounding levels disabled. DB-LIO (DB OFF) achieved an ATE RMSE of 0.296 m—comparable to DB-LIO (DB ON, 0.305 m)—while consuming 2888 MB of memory. This confirmed two key points. First, the accuracy gain over LIO-SAM and Point-LIO was primarily attributed to the enhanced loop closure pipeline incorporating Scan Context-based place recognition and robust noise modeling, not to the DB module itself. Second, and more critically for the central claim of this work, **the DB-based memory bounding mechanism preserved trajectory accuracy despite offloading 81.9% of keyframe data to disk** (ATE RMSE 0.305 m vs. 0.296 m, a difference of only 3%). This near-zero accuracy cost demonstrated that the optimization-aware spatial caching design—which guarantees that all keyframes relevant to pending loop closure and graph optimization reside in cache—successfully prevented the accuracy degradation that would otherwise result from aggressive memory reduction.

### 5.7. Comparison with RTAB-Map (Bounded-Memory Baseline)

RTAB-Map [7] is one of the few widely adopted SLAM systems with built-in memory management, making it the most relevant point of comparison for the bounded-memory claim. Both systems appear in the memory comparison of Table 4 and the accuracy comparison of Table 9; this subsection analyzes the two together under identical sensor and dataset conditions (Livox Mid360, orchard dataset, 37.5 min), and, in particular, explains the large ATE gap (0.305 m vs. 11.166 m) observed in Table 9. For practical reasons (RTAB-Map’s tight ROS 2 launch coupling on our setup), the RTAB-Map run was conducted on the Jetson AGX Orin, whereas the DB-LIO row in Table 4 is a desktop measurement; the comparison is therefore not strictly platform-controlled, but the conclusions below concern the eviction-policy mechanism, which is platform-independent.

*Memory management effectiveness.* Both systems successfully bounded their working set: RTAB-Map capped its working memory at approximately 106 nodes via temporal WM/LTM transfer, while DB-LIO bounded its LRU cache at C=100 keyframes via spatial eviction. In terms of overall memory, RTAB-Map consumed 812 MB (21.7 MB/min), whereas DB-LIO consumed 524 MB (14.0 MB/min) (Table 4), indicating that DB-LIO achieved a lower total memory footprint under equivalent conditions.

*Downstream SLAM quality and eviction policy.* Despite comparable memory bounding, trajectory accuracy diverged sharply (ATE RMSE 0.305 m vs. 11.166 m; Table 9, Figure 7f). Two co-dependent factors explain this gap: RTAB-Map’s bag-of-words (BoW) place recognition detected zero loop closures in the pure LiDAR setting, and its temporal eviction discards nodes by recency regardless of spatial relevance; upon revisiting a mapped area, relevant keyframes may have already been moved to LTM and be unavailable for optimization. DB-LIO’s spatial eviction guarantees that all keyframes within $r_{q}$ reside in cache (Theorem 1), preserving optimization quality. These results validate the distinctions in Table 1: in LiDAR SLAM, *where* a keyframe is located matters more than *when* it was created. Notably, this comparison evaluates RTAB-Map outside its primary design domain (visual SLAM); the observation is therefore less a critique of RTAB-Map than a demonstration that the *choice of eviction policy* is critical when the sensor mode is LiDAR.

### 5.8. Localization Mode Validation (KITTI)

Section 5.2, Section 5.3, Section 5.4, Section 5.5, Section 5.6 and Section 5.7 validated the memory-bounding contribution on the orchard dataset. This subsection turns to the second experimental role: validating the *localization mode* contribution—map reuse without remapping via the DB-based persistent storage—on the KITTI odometry benchmark [34]. The sensor (Velodyne HDL-64E, Velodyne Lidar Inc., San Jose, CA, USA, mechanical 64-channel) and environment (urban driving) differ substantially from the Livox Mid360 orchard setting, thereby also testing generalization of the overall pipeline.

#### 5.8.1. Experimental Design

Using three sequences (00, 05, 07) from the KITTI odometry dataset, we conducted experiments in two stages:

1. **Mapping stage**: Ran DB-LIO in the mapping mode to build a map for each sequence and saved it as an SQLite DB with PCD files.
2. **Localization stage**: Restarted the system from scratch, loaded only the saved DB, and ran in localization mode on the same sequences.

This two-stage design tested both capabilities. The mapping stage validated that DB-LIO built accurate maps (mapping vs. GT (MvG)), while the localization stage verified that the system could achieve stable pose estimation solely through DB-based map matching without generating new keyframes.

#### 5.8.2. Results and Analysis

**Figure 8:**
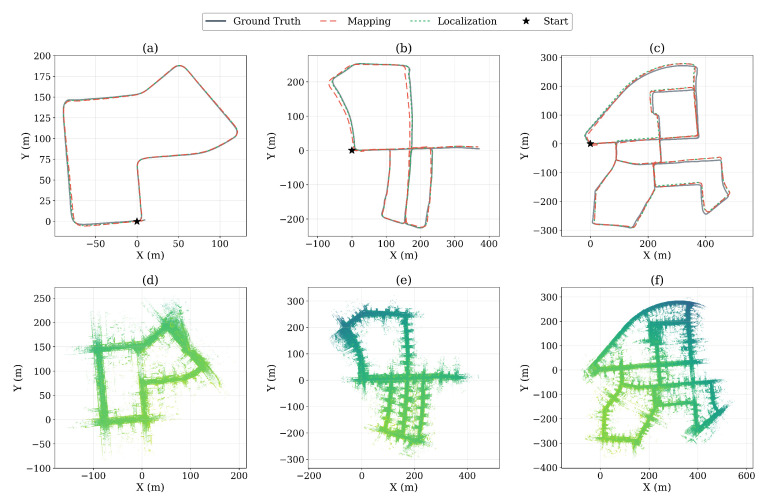
KITTI localization mode results. (**a**–**c**) Trajectory comparison (ground truth, mapping, and localization) for sequences 07, 05, and 00, respectively. (**d**–**f**) Reconstructed point cloud maps for each sequence, colored by height.

Table 10 and Figure 8 show the results of the mapping and localization modes on three KITTI sequences.

As shown in Table 10, the localization mode (LvG) achieved an average ATE of 2.05 m, nearly matching the mapping mode (MvG, 1.89 m). The focus of this experiment was the *consistency* between the two modes rather than absolute accuracy. Notably, mapping vs. localization (MvL) averaged 0.70 m, demonstrating that the localization mode generated nearly identical trajectories to the mapping mode. In **seq05**, MvL was 0.23 m, showing virtually identical trajectories between the two modes. This confirmed that stored DB keyframes and Scan Context-based global matching operated reliably.

Because the localization mode did not generate new keyframes, memory usage remained strictly constant at the O(C) level established by the initial DB load. As shown in the Loc. RSS column of Table 10, the localization RSS stabilized within 364–488 MB across all three sequences regardless of the stored map size, confirming bounded memory behavior on a public benchmark. These results showed that DB-LIO was not merely a memory optimization technique but a complete SLAM framework that eliminated remapping costs in environments requiring repeated operation. For agricultural robots that repeatedly traversed the same orchard paths or logistics robots operating in fixed warehouse layouts, a single mapping session produced a DB that could be reused indefinitely.

### 5.9. Embedded Platform Validation (Jetson AGX Orin)

The preceding subsections established memory bounding, accuracy, and localization capability on a desktop PC under the three-level bounding configuration. This final subsection closes the experimental evaluation by validating the complete four-level architecture on the target embedded platform, the NVIDIA Jetson AGX Orin (ARM Cortex-A78AE, 32 GB LPDDR5, NVMe SSD), using the same orchard dataset. The Jetson run additionally enabled the iSAM2 sliding window compaction (Section 4.1.7), allowing simultaneous validation of the fourth bounding level and of on-robot deployability.

Table 11 compares the key memory metrics between the two platforms.

**Figure 9:**
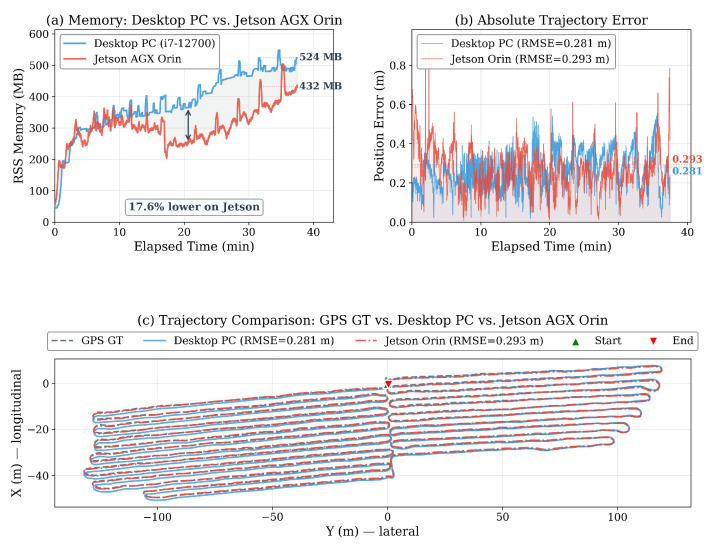
Embedded platform validation on Jetson AGX Orin (all four bounding levels active). (**a**) RSS memory comparison between the desktop PC (3-level, i7-12700) and the Jetson AGX Orin (4-level) over 37.5 min. The Jetson achieved 17.6% lower final RSS owing to the additional iSAM2 compaction and ARM allocator characteristics. (**b**) Absolute trajectory error over time; both platforms achieved near-identical accuracy (RMSE 0.305 m vs. 0.293 m). (**c**) Trajectory comparison against GPS ground truth, confirming equivalent SLAM quality on both platforms.

The Jetson AGX Orin achieved a 17.6% lower final RSS (432 MB vs. 524 MB) and a 36.8% lower post-saturation growth rate (4.9 MB/min vs. 7.7 MB/min) compared with the desktop PC. This improvement was attributed to two factors: (i) the iSAM2 sliding window compaction, active only in the Jetson experiment, periodically rebuilt the Bayes tree with only the most recent $W_{\mathrm{keep}}$ poses, producing the sharper memory drops visible in Figure 9a; and (ii) the ARM memory allocator’s more aggressive page reclamation, which reduced heap fragmentation compared with the x86 glibc allocator.The compaction sawtooth pattern in the Jetson curve confirmed that the fourth bounding level operated as designed (Section 4.1.7), actively reclaiming the Bayes tree memory that remained unbounded in the three-level desktop configuration. Over the 37.5 min run, approximately 21 compaction events were observed (counted as RSS drops greater than 15 MB in the Jetson memory trace), providing direct empirical evidence that the fourth bounding level fired repeatedly rather than only at run-end. Trajectory accuracy was also preserved: the Jetson achieved an ATE RMSE of 0.293 m, nearly identical to the desktop’s 0.305 m (a difference of only 0.012 m, within the noise margin of GPS ground truth). As shown in Figure 9b,c, the error profiles and estimated trajectories were visually indistinguishable between the two platforms. The number of detected loop closures was nearly identical (2338 vs. 2339), confirming that the SLAM pipeline operated equivalently on both architectures. These results demonstrated that the full four-level O(C) memory bounding of DB-LIO held on the embedded ARM platform, yielding a lower memory footprint than the three-level desktop configuration while maintaining equivalent trajectory accuracy; this validates the system’s suitability for deployment on memory-constrained embedded robots. When read in conjunction with the desktop results above, these findings confirm that the desktop measurements represent a conservative upper bound on the memory footprint expected in the full four-level deployment.

## 6. Discussion and Limitations

While DB-LIO demonstrated effective memory bounding without sacrificing accuracy, several limitations and design trade-offs merit discussion.

**Cold Start Latency.** When the system was launched in the localization mode, the LRU cache was initially empty. Until the active window was populated through spatial queries and disk loads, the first few seconds of operation could experience elevated cache miss rates and correspondingly higher I/O latency. In practice, this cold start phase was limited to the time required to load *C* keyframes from the disk (at most C×0.12ms≈12ms for C=100 on NVMe SSD), which was negligible relative to the robot’s startup sequence.

**Disk I/O on Different Storage Media.** The memory savings of DB-LIO came at the cost of disk storage and I/O. To evaluate the impact of slower storage on real-time performance, the SLAM-specific I/O workload was benchmarked on both the NVMe SSD and the internal eMMC of the Jetson AGX Orin using actual keyframe data (1391 PCD files, averaging 29 KB each). Table 12 summarizes the results.

Although the raw I/O throughput of eMMC was 3–15× slower than that of NVMe (244 vs. 2154 MB/s sequential read; 127 vs. 1962 MB/s sequential write), the impact on SLAM real-time performance was tolerable. Applying the same worst-case scenario as in Section 5.4 (a loop-closure batch with 10 cache misses), on NVMe this totaled 10×0.12=1.2 ms (1.2% of the 100 ms frame budget), whereas on eMMC the same batch cost 10×3.0=30 ms (30%), owing to the slower per-load latency. For a single isolated cache miss, the corresponding figures were 0.12 ms (NVMe) versus 3.3 ms (eMMC, including the 296 μs spatial query). Because the loop closure module operated asynchronously in a separate thread from the main LIO odometry, even the eMMC worst case did not stall pose estimation; the LRU cache further ensured that cache misses were rare during steady-state operation, and keyframe writes were asynchronous and infrequent. The full map load at startup took 175 ms on eMMC, an acceptable one-time cost. These results confirmed that DB-LIO’s disk-backed architecture maintained real-time performance even on the slower embedded storage medium. Additionally, while memory was bounded at O(C), disk usage grew as O(N); periodic pruning or lossy compression could manage disk growth in prolonged deployments.

**Residual Memory Growth.** As formalized in Equation (14), the total memory consists of a dominant O(C) term (dense data: point clouds, cache, descriptors, Bayes tree) and a secondary $O(N_{\mathrm{sparse}})$ term (metadata and overhead). A residual growth rate of 7.7 MB/min was observed after cache saturation (from approximately 3 min onward; Section 5.3). Crucially, this residual growth did not originate from the three active bounded O(C) components (keyframe trimming, LRU cache, descriptor pool). The visible memory drops in the desktop experiment were attributed to LRU cache eviction and subsequent heap reclamation via malloc_trim. The fourth bounding level—iSAM2 sliding window compaction—was validated separately on the Jetson AGX Orin (Section 5.9), where all four levels operated simultaneously and achieved a 17.6% lower final RSS, confirming that the Bayes tree bounding provided additional memory savings beyond the three-level desktop configuration.

To clarify the source of the residual growth, a per-component memory breakdown analysis was performed. Table 13 quantifies the estimated memory contribution of each ancillary component at the end of the 37.5 min run (5867 keyframes).

As shown in Table 13, the anchor graph and loop history contributed negligibly (<1 MB combined). The dominant source of residual growth was heap fragmentation: repeated cache eviction and point cloud reloading created memory fragments that the allocator did not immediately return to the operating system, even after explicit malloc_trim calls. This is a well-known behavior of the glibc memory allocator in long-running processes with frequent allocation cycles and does not represent a leak in the O(C) bound on the four primary memory consumers. The ROS 2 middleware contributed approximately constant overhead from message queues and TF buffers.

In practical terms, even with this residual overhead, the projected memory remained substantially lower than that of LIO-SAM under an equivalent operation time, staying well within the capacity of the target platform (Jetson AGX Orin, 32 GB LPDDR5). Adopting a custom memory pool allocator for point cloud buffers to eliminate fragmentation, bounding the anchor graph via periodic pruning, and capping the loop history size are directions for future work that would bring the practical memory curve closer to the theoretical O(C) bound.

**Experimental Scope and Persistent Operation.** The term “persistent mapping” in this work carries a dual meaning: (i) bounded memory during a single extended mapping session, and (ii) the ability to store, reload, and reuse previously built maps for localization, as demonstrated by the KITTI localization experiments (Section 5.8). The primary memory bounding results were derived from a 37.5 min run on the custom orchard dataset. Although longer continuous runs would further validate the bounded memory behavior, the residual growth (7.7 MB/min) was attributable to heap fragmentation and middleware overhead rather than architectural leakage, as all four bounded components remained at O(C) throughout the experiment (see Table 13). The orchard’s repetitive, corridor-like structure may artificially inflate the cache hit rate; validation on large-scale, non-repetitive urban and campus datasets is planned as future work.

## 7. Conclusions

This study presented DB-LIO, a database-driven LiDAR–inertial odometry system that addresses unbounded memory growth in 3D LiDAR SLAM by tightly coupling spatial cache management with factor-graph optimization; its core idea—optimization-aware spatial caching—aligns the cache eviction boundary with the loop closure search radius via R-Tree spatial indexing, guaranteeing that every keyframe relevant to the next graph optimization cycle remains in memory, and the four-level bounding architecture that realizes this design confines the dominant memory consumers to O(C) with only an $O(N_{\mathrm{sparse}})$ residual. On a 37.5 min orchard run, DB-LIO reduced memory by 81.9% (2888 MB → 524 MB) at a near-zero accuracy cost (ATE RMSE 0.305 m vs. 0.296 m) and only 0.12 ms average I/O overhead; against the bounded-memory baseline RTAB-Map (812 MB, ATE 11.166 m), DB-LIO achieved both a smaller footprint and far better SLAM quality, illustrating that in LiDAR SLAM *where* a keyframe is located matters more than *when* it was created. The DB-backed persistent storage further enables map reuse without remapping: on KITTI sequences 00, 05, and 07 the localization mode attained an average LvG ATE of 2.05 m and an MvL of 0.70 m while RSS remained bounded within 364–488 MB regardless of map size, and on the target embedded platform (Jetson AGX Orin), activating all four bounding levels produced a 17.6% lower footprint (432 MB) than the three-level desktop configuration, with trajectory accuracy preserved within 0.012 m of the desktop value, confirming practical viability for memory-constrained robots. **Limitations and future work.** The O(C) bound applies to the four primary memory consumers under the spatial density assumption of Theorem 1; the residual growth terms (the $O(N_{\mathrm{sparse}})$ anchor graph and loop history, glibc heap fragmentation, and degraded operation when $\rho \;>\;C_{\mathrm{db}}$ is sustained) are quantified in Section 6 and Table 13, and the primary validation was performed on a single 37.5 min orchard dataset whose corridor-like, repetitive structure favors the spatial caching strategy. Beyond the custom memory-pool allocator and anchor-graph pruning already noted in the Discussion, future work targets an adaptive online policy that tunes $C_{\mathrm{db}}$ and $r_{q}$ from observed local keyframe density (removing the need for manual environment-specific tuning), validation on larger and more diverse benchmarks (urban driving, campus, multi-floor indoor), and extension of the persistent DB to support shared-database multi-robot mapping with concurrent spatial queries.

## Figures and Tables

**Table 1:** Methodological comparison of memory management approaches in SLAM systems.

Criterion	Cartographer	RTAB-Map	DB-LIO
Eviction policy	Submap freezing	Temporal (node count)	Spatial (R-Tree)
Optimization-aware	No	No	Yes
Spatial indexing	Grid-based	Bag-of-words	R-Tree
Data retained	Compressed grid	Visual features	Full point cloud
Map reuse	Limited	Yes (visual)	Yes (LiDAR)

**Table 2:** Key implementation parameters.

Module	Parameter	Value
active window	Radius	50 m
	Cache Size ($W=C_{\mathrm{db}}$)	100 frames
Scan Context	Max Pool Size ($S_{\max}$)	2000
Graph Compaction	Window Max Size	500 poses
	Window Keep Size	200 poses
Loop Closure	Search Radius ($r_{lc}$)	10 m
	ICP Fitness Threshold	0.1
	Max Rotation	60^∘^
Loop Factor Noise	Cauchy *k*/Diag. $\sigma^{2}$	1.0/0.3

**Table 3:** Memory complexity of compared SLAM systems.

System	Complexity	Management Method
LIO-SAM [2]	O(N)	All keyframes in-memory
FAST-LIO2 [3]	$O(N_{\mathrm{pts}})$	ikd-Tree full map
Cartographer [6]	O(S)	Submap-based
RTAB-Map [7]	$O(W_{\max})$	WM/LTM hierarchy
**DB-LIO**	O(C)	Four-level bounding

**Table 4:** Memory usage comparison (custom dataset, resident set size (RSS)-based). The **Platform** column indicates the execution hardware for each row; see Section 5.1 and footnotes for the cross-platform RTAB-Map rationale.

System	Platform	Duration	RSS Final	Growth Rate	Note
**DB-LIO** ^¶^	Desktop PC	37.5 min	**524 MB**	**14.0 MB/min**	Bounded-rate
LIO-SAM	Desktop PC	37.5 min	733 MB	19.6 MB/min	Linear growth
Point-LIO	Desktop PC	37.5 min	1329 MB	35.4 MB/min	Linear growth
DLIO	Desktop PC	6.5 min ^†^	4510 MB	698.6 MB/min	OOM stop
FAST-LIO2	Desktop PC	25.4 min ^‡^	9701 MB	382.3 MB/min	Diverged
RTAB-Map ^§^	Jetson AGX Orin	37.5 min	812 MB	21.7 MB/min	WM/LTM bounded

Bold values indicate the proposed method (DB-LIO) and its best-in-class results. Growth Rate: RSS Final/Duration. Same sensor (Livox Mid360) and dataset across both platforms. ^§^ Bounded memory via WM/LTM architecture; see Section 5.7 for full discussion of the platform choice and comparison. ^¶^ Three-level bounding (without iSAM2 compaction); four-level result: 432 MB on Jetson (Section 5.9). ^†^ Early termination owing to out of memory (OOM). ^‡^ Early termination owing to divergence.

**Table 5:** DB mode ON/OFF comparison: memory and accuracy. RPE denotes relative pose error.

DB	Final RSS	ATE RMSE	RPE	Memory
Mode	(MB)	(m)	(m)	Reduction
OFF	2888	0.296	0.114	–
**ON**	**524**	**0.305**	**0.108**	**81.9%**

**Table 6:** Cache performance and disk usage (custom orchard dataset).

Hit Rate	Avg Load	Disk	Keyframes
(%)	(ms)	(MB)	
90.8	0.12	166	5867

**Table 7:** Ablation: Cache size *C* (active window radius = 50 m).

Cache *C*	RSS (MB)	ATE (m)	Hit Rate (%)	Reduction
25	361	0.408	65.5	87.5%
50	413	0.326	83.0	85.7%
100	524	0.305	90.8	81.9%
200	1754	0.293	94.0	39.3%
*∞* (OFF)	2888	0.296	100	–

**Table 8:** Ablation: active window radius $r_{q}$ (cache size = 100).

Radius (m)	RSS (MB)	ATE (m)	Hit Rate (%)	Avg Load (ms)
30	512	0.313	90.8	0.12
50	524	0.305	90.8	0.12
100	518	0.311	90.7	0.12

**Table 9:** Trajectory accuracy comparison (custom dataset, GPS reference, time: min, ATE/RPE: m).

System	Time	RMSE	Mean	Max	RPE	Note
**DB-LIO**	37.5	0.305	0.290	0.917	0.108	-
DB-LIO (DB OFF)	37.5	0.296	0.281	0.919	0.114	2888 MB
LIO-SAM	37.5	0.508	0.488	1.130	0.139	-
Point-LIO	37.5	0.516	0.480	1.322	0.129	-
RTAB-Map	37.5	11.166	8.559	27.864	3.244	LiDAR-only ^§^

^§^ RTAB-Map ran in its native 3D LiDAR configuration; the large ATE/RPE reflect its visual-centric pipeline operating without camera input.

**Table 10:** KITTI per-sequence Mapping vs. Localization mode comparison (ATE unit: m, RSS unit: MB). MvG: Mapping vs. GT, LvG: Localization vs. GT, MvL: Mapping vs. Localization (trajectory consistency between modes). Loc. RSS: Localization mode final RSS memory.

Seq	Frames	Path (m)	MvG	LvG	MvL	Loc. RSS
07	1106	695.0	0.73	0.84	**0.42**	364
05	2762	2205.1	1.78	1.75	**0.23**	481
00	4544	3724.9	3.16	3.57	1.46	488
**AVG**	-	-	1.89	2.05	0.70	444

**Table 11:** Platform comparison: Desktop PC (3-level) vs. Jetson AGX Orin (4-level, with iSAM2 compaction). Same dataset, 37.5 min.

Platform	Final RSS	Peak RSS	Post-Sat. Rate	ATE RMSE	Loops
Desktop PC (i7-12700)	524 MB	549 MB	7.7 MB/min	0.305 m	2339
**Jetson AGX Orin**	**432 MB**	**504 MB**	**4.9 MB/min**	**0.293 m**	**2338**
**Difference**	**−17.6%**	**−8.2%**	**−36.8%**	−0.012 m	<0.1%

Post-Sat. Rate: RSS growth rate after cache saturation (from 3 min onward).

**Table 12:** I/O latency comparison: NVMe SSD vs. eMMC on Jetson AGX Orin.

SLAM Operation	NVMe	eMMC	% of 10 Hz Budget
R-Tree spatial query	59 μs	296 μs	0.3%
Keyframe PCD load (29 KB)	2.6 ms	3.0 ms	3.0%
Keyframe write + fsync	63 μs	770 μs	0.8%
Full map load (39 MB)	55 ms	175 ms	One-time

Budget %: eMMC latency relative to 100 ms (10 Hz LiDAR frame period).

**Table 13:** Per-component memory breakdown of ancillary (unbounded) structures after 37.5 min.

Component	Growth Model	Estimated Size
Sparse global anchor graph	$O(N/A_{\mathrm{int}})$, $A_{\mathrm{int}}=100$	58×48 B ≈ 2.8 KB
Loop closure history	O(L), *L* = detected loops	∼0.5 MB
Heap fragmentation	Process-dependent	∼150–200 MB
ROS 2/middleware buffers	Approximately constant	∼50–80 MB
**Total residual**		∼200–280 MB

## Data Availability

The source code of DB-LIO (v1.0) will be made publicly available on GitHub upon acceptance of this paper. The custom orchard dataset used in this study is available from the authors upon reasonable request. The KITTI odometry benchmark is publicly available at https://www.cvlibs.net/datasets/kitti/ (accessed on 10 May 2026).

## Abbreviations

The following abbreviations are used in this manuscript:

SLAM	Simultaneous Localization and Mapping
LIO	LiDAR-Inertial Odometry
OOM	Out-Of-Memory
LRU	Least Recently Used
ATE	Absolute Trajectory Error
RPE	Relative Pose Error
RSS	Resident Set Size
DB	Database
PCD	Point Cloud Data
